# *Corynebacterium ulcerans* in Ferrets

**DOI:** 10.3201/eid2001.130675

**Published:** 2014-01

**Authors:** Robert P. Marini, Pamela K. Cassiday, Jaime Venezia, Zeli Shen, Ellen M. Buckley, Yaicha Peters, Nancy Taylor, Floyd E. Dewhirst, Maria L. Tondella, James G. Fox

**Affiliations:** Massachusetts Institute of Technology, Cambridge, Massachusetts, USA (R.P. Marini, J. Venezia, Z. Shen, E.M. Buckley, Y. Peters, N. Taylor, J.G. Fox);; Centers for Disease Control and Prevention, Atlanta, Georgia, USA (P.K. Cassiday, M.L. Tondella);; The Forsyth Institute, Cambridge (F.E. Dewhirst);; Harvard School of Dental Medicine, Boston, Massachusetts, USA (F.E. Dewhirst)

**Keywords:** Corynebacterium ulcerans, ferret, cephalic implant, bacteria, United States, Massachusetts, Mustela putorius furo

**To the Editor:** Infection with *Corynebacterium ulcerans* occurs sporadically throughout the world, and in the United Kingdom it has emerged as the most common cause of diphtheria-like disease (*1*). *C. ulcerans*, along with *C. diphtheriae* and *C. pseudotuberculosis*, can be lysogenized by diphtheria toxin–encoding bacteriophages; this process enables the organism to induce its characteristic sequela (the diphtheritic membrane) in the host. *C. ulcerans* in the environment has been a source of mastitis in cattle and a cause of diphtheria in humans who consume unpasteurized, contaminated milk. The organism has been isolated from various domestic, wild, and laboratory animals; additional definitive sources are dogs, cats, and pigs (*2*)*.* *C. ulcerans* has been isolated from bonnet macaques with mastitis and from the cephalic implants of purpose-bred macaques used in cognitive neuroscience experiments (*3**,**4*)*.* We report isolation of *C. ulcerans* from cephalic implants in 4 ferrets (*Mustela putorius furo*) and the oropharynx of 1 ferret, all used in imaging experiments in Massachusetts, USA, during 2007–2008.

All ferrets described here were purpose-bred, domestic ferrets, purchased from a commercial vendor. The index case occurred in a ferret with a cephalic implant. Microbiological culture of a purulent discharge from the implant margin yielded a polymicrobial infection that included an organism identified as *C. ulcerans* by the API Coryne strip system (bioMérieux, Durham, NC, USA) (Table). This isolate and additional isolates from mixed infections of the implants of 3 other ferrets were subsequently identified as *C. ulcerans* by our diagnostic laboratory (Division of Comparative Medicine, Massachusetts Institute of Technology, Cambridge, MA, USA) and by the Centers for Disease Control and Prevention (Atlanta, GA, USA) by use of the API Coryne strip system. The oropharyngeal isolate was originally identified by our laboratory as *C. pseudotuberculosis* (99.5%); the same test performed at the Centers for Disease Control and Prevention yielded ambiguous results (*C. ulcerans* [87.3%] and *C. pseudotuberculosis* [12.5%]).

**Table:** Identification of *Corynebacterium ulcerans* strains isolated from ferrets*

MIT accession no.	CDC API code	CDC interpretation (confidence limit, %)	Isolate source	CDC MALDI-TOF-MS (score)†	16S rRNA (confidence limit, %), GenBank accession no.	*rpoB *(confidence limit, %), GenBank accession no.
07–3331	0101326‡	*C. ulcerans* (87.3), *C. pseudotuberculosis* (12.5)	Oropharynx	*C. ulcerans* (2.13)	*C. ulcerans* (99.5) KF564646	*C. ulcerans* (99.5) KF539859
08–0584	0111326	*C. ulcerans* (99.7)	Cephalic implant	*C. ulcerans* (2.35)	*C. ulcerans* (99.5) KF564647	*C. ulcerans* (99.5) KF539860
07–3276	0111326	*C. ulcerans* (99.7)	Cephalic implant	*C. ulcerans* (2.24)	*C. ulcerans* (99.7) KF564645	*C. ulcerans* (99.5) KF539858

*Identification was performed by use of the API Coryne strip system (bioMérieux, Durham, NC, USA), MALDI-TOF-MS (matrix-assisted laser desorption–ionization time-of-flight mass spectroscopy) (Bruker Daltonics; Fremont, CA, USA), and gene sequencing. Percentages after the API identification refer to confidence limits. Percentages after the gene sequencing results refer to percentage identities with a reference strain (*4*). MIT, Massachusetts Institute of Technology; CDC, Centers for Disease Control and Prevention. †2.000–2.299, secure genus identification, probable species identification; 2.300–3.000, highly probable species identification (*4*). ‡The API code generated at MIT was 0101320, interpreted as *C. pseudotuberculosis* (99.5%).

Three isolates (2 implant isolates and the oropharyngeal isolate) were subsequently characterized by MALDI-TOF-MS (matrix-assisted laser desorption–ionization time-of-flight mass spectroscopy) Bruker Daltonics, Fremont, CA, USA) and by 16S rRNA sequencing (*5*) and partial *rpoB* (*6*) gene sequencing (Table). The conserved primers C2700F and C3130R from the *rpoB* gene were used to amplify the PCR products (*6*)*.* All were confirmed to be *C. ulcerans*. The presence of toxin genes for diphtheria toxin (*tox*) (*7*) and phospholipase D (*pld*) (*3*) were evaluated by PCR. Diphtheria toxin production was evaluated by a modified Elek test (*4*)*.* None of the isolates produced diphtheria toxin or contained the diphtheria toxin gene; all isolates were phospholipase-D positive for the 720-bp product.

To determine the source of the isolates, we tested the ferret isolates along with 3 select isolates from our macaque colony by BOX PCR and random amplified polymorphic DNA analysis. Neither type of analysis of the ferret and macaque *C. ulcerans* strains identified any common patterns (data not shown). Ferrets and macaques were housed in separate rooms in the same vivarium; animal care technicians were dedicated to 1 of the 2 species during any particular month. The prevalence of *C. ulcerans* in our macaque population and the precedence of its isolation from those animals more than a decade ago strongly suggests that the isolates are of macaque origin (*3**,**4*)*.* More exhaustive comparison of the ferret isolates with archived macaque isolates might provide a match. The possibility also exists that newly acquired ferrets arrived infected with *C. ulcerans* or contracted it from an animal technician, veterinarian, or researcher. These possible sources of *C. ulcerans* infection have not been investigated.

An organism recently isolated from the lung, liver, and kidney tissue of a ferret that died of sepsis has been designated as a novel species, *C. mustelae* (*8*)*. C. mustelae* is 96.78% related to *C. ulcerans* in 16S rRNA gene sequence similarity and is the first member of the genus to be implicated in disease of ferrets. *C. ulcerans* must now also be considered a potential pathogen of ferrets, although the mixed nature of these implant infections precludes definitive etiologic statements. Implant infection and oropharyngeal carriage in ferrets potentially represent additional zoonotic sources of this organism, underscoring the need for accurate and complete characterization of coryneform bacteria. Notably, the API Coryne test was unable to definitively identify the oropharyngeal isolate, a result reported by our group for other studies and by other investigators (*4*)*.* The results of additional characterization modalities were all concordant. The *C. ulcerans* isolates from this study were nontoxigenic, and their potential for causing classical diphtheria is unlikely (Table). In contrast, a non–diphtheria toxin–producing *C. ulcerans* skin infection mimicking cutaneous diphtheria in a 29-year-old man was recently reported (*9*)*.* Although the source of *C. ulcerans* was not definitively determined, nontoxigenic *C. ulcerans* was later isolated from the oral cavity of the patient’s pet cat. Identity of these 2 isolates was not confirmed by molecular identification techniques (*9*). In another case, strain identity was established between a toxigenic isolate cultured from a woman with clinical diphtheria and the same organism cultured from her asymptomatic cat (*2*). Toxigenic and nontoxigenic isolates of *C. diphtheriae* have been reported to cause the cutaneous form of this disease (*10*).

## References

[1] Wagner KS, White JM, Crowcroft NS, De Martin S, Mann G, Efstratiou A. Diphtheria in the United Kingdom, 1986–2008: the increasing role of *Corynebacterium ulcerans.* Epidemiol Infect. 2010;138:1519–30 and. 10.1017/S0950268810001895 20696088

[2] Berger A, Huber I, Merbecks S-S, Konrad R, Hormansdorfer S, Hogardt M, Toxigenic *Corynebacterium ulcerans* in woman and cat. Emerg Infect Dis. 2011;17:1767–9 . 10.3201/eid1709.110391 21888821 PMC3322090

[3] Bergin IL, Chien C-C, Marini RP, Fox JG. Isolation and characterization of *Corynebacterium ulcerans* from cephalic implants in macaques. Comp Med. 2000;50:530–5 . 11099137

[4] Venezia J, Cassiday PK, Marini RP, Shen Z, Buckley EM, Peters Y, Characterization of *Corynebacterium* species in macaques. J Med Microbiol. 2012;61:1401–8. 10.1099/jmm.0.045377-0 22723254 PMC3541768

[5] Dewhirst FE, Chen T, Izard J, Paster BJ, Tanner ACR, Yu W-H, The human oral microbiome. J Bacteriol. 2010;192:5002–17. 10.1128/JB.00542-10 20656903 PMC2944498

[6] Khamis A, Raoult D, La Scola B. *rpoB* gene sequencing for identification of *Corynebacterium* species. J Clin Microbiol. 2004;42:3925–31. 10.1128/JCM.42.9.3925-3931.2004 15364970 PMC516356

[7] Schuhegger R, Lindermayer M, Kugler R, Heesemann J, Busch U, Sing A. Detection of toxigenic *Corynebacterium diphtheriae* and *Corynebacterium ulcerans* strains by a novel real-time PCR. J Clin Microbiol. 2008;46:2822–3. 10.1128/JCM.01010-08 18550743 PMC2519500

[8] Funke G, Frodi R, Bernard KA. *Corynebacterium mustelae* sp. nov., isolated from a ferret with lethal sepsis. Int J Syst Evol Microbiol. 2010;60:871–3. 10.1099/ijs.0.010942-0 19661496

[9] Corti MAM, Bloemberg GV, Borelli S, Kutzner H, Eich G, Hoelzle L, Rare human skin infection with *Corynebacterium ulcerans*: transmission by a domestic cat. Infection. 2012;40:575–8. 10.1007/s15010-012-0254-5 22403045

[10] Gordon CL, Fagan P, Hennessy J, Baird R. Characterization of *Corynebacterium diphtheriae* isolates from infected skin lesions in the Northern Territory of Australia. J Clin Microbiol. 2011;49:3960–2 . 10.1128/JCM.05038-11 21918025 PMC3209100

